# Development and Validation of Nutrition Literacy Assessment Instrument for Chinese Lactating Women: A Preliminary Study

**DOI:** 10.3390/nu15153488

**Published:** 2023-08-07

**Authors:** Zhao Li, Yalin Zhou, Yuwei Tan, Xiaoyu Zhu, Wei Liu, Yuhan Chen, Yong Qin, Ruijun Li, Lanlan Yu, Runlong Zhao, Yajun Xu

**Affiliations:** 1 School of Public Health, Peking University, No. 38 Xueyuan Road, Beijing 100083, China; 1810306115@pku.edu.cn (Z.L.); 2216393053@bjmu.edu.cn (Y.Z.); 1911110168@bjmu.edu.cn (Y.T.); 2211110219@bjmu.edu.cn (X.Z.); 1511210188@bjmu.edu.cn (W.L.); 1410306130@bjmu.edu.cn (Y.C.); 1310306125@bimu.edu.cn (Y.Q.); 1811210224@bjmu.edu.cn (R.L.); yulanlan_pucri@bjmu.edu.cn (L.Y.); 1510306204@pku.edu.cn (R.Z.); 2 Beijing Key Laboratory of Toxicological Research and Risk Assessment for Food Safety, Peking University, No. 38 Xueyuan Road, Beijing 100083, China; 3 PKUHSC—China Feihe Joint Research Institute of Nutrition and Healthy Lifespan Development, No. 38 Xueyuan Road, Beijing 100083, China

**Keywords:** nutrition literacy, assessment instrument, lactating women

## Abstract

This study focused on the development and validation of a nutrition literacy assessment instrument for Chinese lactating women (NLAI-L). A comprehensive literature review and group discussion by experts in relevant fields were adopted to determine the dimension, topics and questions of NLAI-L. Content validity was evaluated by a panel of experts. The exploratory factor analyses (EFA) and confirmatory factor analyses (CFA) were used to evaluate the construct validity. Cronbach’s α and split-half reliability were applied to examine the reliability of NLAI-L. The final NLAI-L consisted of 38 questions covering three dimensions: knowledge, behavior and skill. The EFA revealed four sub-domains for knowledge, one sub-domain for behavior and four sub-domains for skill. The results showed that NLAI-L had satisfactory content validity (CVI = 0.98, CVR = 0.96), good reliability (Cronbach’s α coefficient = 0.84) and acceptable construct validity (χ^2^/*df* = 2.28, GFI = 2.81, AGFI = 0.79, RMSEA = 0.057). In the application part, the average NL score was 46.0 ± 9.3. In multivariate linear regression, education level, age, postnatal period and occupation were the potential influencing factors of NL for Chinese lactating women. The study established an effective and reliable assessment instrument for Chinese lactating women (NLAI-L) through qualitative and quantitative methods. The establishment of NLAI-L will provide an effective tool for exploring the role of NL in health or disease and provide a basis for the formulation of targeted nutrition interventions.

## 1. Introduction

Health literacy (HL) refers to the ability of individuals to obtain, understand and process basic health information or services and to make correct health decisions [1]. The level of HL is related to the health status of individuals and even society as a whole. At present, HL has become the core goal of international public health. Improving HL plays a key role in improving the overall health level of the population, the utilization rate of preventive health services, reducing hospitalization rates and strengthening disease prevention and control. In 2008, the Ministry of Health of China issued 66 items of Chinese Citizens’ Health Literacy—basic knowledge and skills, which is an official document to elaborate on the content of citizens’ basic health literacy. Continuous monitoring of HL has been carried out throughout China.

Nutrition literacy (NL) is an important part of HL that refers to the ability of individuals to obtain, understand and process nutritional information or services and to use information and services to make correct decisions so as to maintain and promote their own health [2]. NL can affect individual food choices and dietary behavior [3], so it is of great significance in promoting health and nutrition-related chronic disease control [4,5].

Lactation is a special physiological stage in which mothers feed infants with breast milk to achieve the best growth and development and lay the foundation for their adult health. At this stage, lactating women not only produce enough breast milk to feed their offspring but also gradually compensate for their physical consumption during pregnancy and delivery, so their energy and nutritional requirements are significantly higher than those of non-lactating women. However, nutrition and health-monitoring data show that Chinese lactating women have a variety of nutritional problems, such as unreasonable dietary structure, insufficient intake of micronutrients, weight retention and so on [6,7,8].

In recent years, studies have pointed out that the lack of NL may be an important cause of nutritional health problems. Improving NL during lactation is the basic measure to promote the health of lactating women. Improving the NL level of lactating women contributes to improving their ability to acquire, apply and identify nutritional knowledge and establishing healthy eating habits and improving feeding skills, which has a positive impact on both mothers and infants. Therefore, improving the NL is an important measure to promote the health status of lactating women, prevent nutrition-related chronic diseases and is conducible to the healthy growth and development of their offspring. The nutrition literacy assessment instrument is an effective method to measure the level of NL. At present, a nutrition literacy assessment instrument for general adults, school-age children and the elderly has been developed at home and abroad, and there is an urgent need to establish an assessment instrument suitable for lactating women. The research group has established a total of 24 core items of NL for Chinese lactating women [9]. Based on this, this study aims to establish and validate the instrument for assessing the NL of Chinese lactating women and then apply it to the target population.

## 2. Materials and Methods

This study was carried out in two phases:

Phase 1 was the establishment of NL core items for lactating women in China.

Phase 2 was conducted for the development, validation and application of NLAI-L.

**Figure 1:**
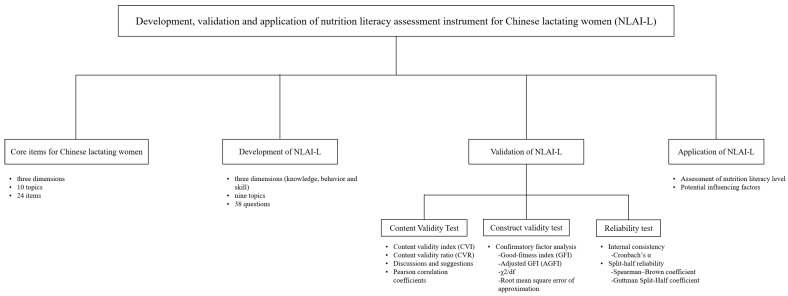
The flow chart of the study on the development, validation and application of nutrition literacy assessment instrument for Chinese lactating women (NLAI-L).

The flow chart of the study is demonstrated in Figure 1.

Based on 66 items from Chinese Citizen’s Health Literacy—basic knowledge and skills, the research group systematically reviewed the literature, combined with the main nutrition-related problems existing during lactation in China, and selected dietary and nutrition-related recommendations for lactating women to form a list of NL items for lactating women. After two rounds of Delphi expert consultation and adjustments, 24 items were selected as core items of NL for lactating women. The core items are divided into three dimensions (basic knowledge and concept, lifestyle and dietary behavior and basic skills) and 10 topics (feeding knowledge, nutrition concept, food and nutrition knowledge, lifestyle, feeding behavior, dietary behavior, supplementary food production, nutrition information acquisition, information identification and decision-making).

The core items were scientific, reliable and had been previously published [9]. The NL core items for Chinese lactating women are shown in Table 1.

### 2.1. The Development of NLAI-L

Based on the NL core items for lactating women mentioned above, the initial NLAI-L was conducted, consisting of three dimensions, eight topics and 54 questions.

A total of 811 questionnaires were collected, of which 793 were valid. Inclusion criteria were age ≥ 18 years old, being able to read and understand words correctly, no mental disorders (diagnosed mental disorders including bipolar disorder, schizophrenia, paranoid mental disorder, etc.), no malignant tumors and willingness to participate in the survey. Exclusion criteria were cognitive dysfunction and incapability of cooperating with the questionnaire survey. Before collecting data, all respondents signed their informed consent. The research was approved by the Medical Ethics Committee of Peking University (IRB00001052-17107).

Respondents were randomly divided into two groups, with the first group (*n* = 396) selected for the development of NLAI-L and the second group (*n* = 397) selected for the validation of NLAI-L. After consultation with experts (two doctoral supervisors majoring in maternal and child nutrition, two master’s degree supervisors, and two doctoral students majoring in nutrition and food hygiene) and selection of questions, the finalized NLAI-L consisted of three dimensions, nine topics under dimensions and a total of 38 questions.

The whole NLAI-L consisted of two parts: The first part was designed to collect the demographic, information such as age, ethnicity, occupation and education level. The second part was the NL questionnaire for Chinese lactating women. The scoring standards of each question were detailed as follows:

The questions included single-choice, multiple-choice questions and Likert-type questions. Single-choice questions were scored 2 points for correct answers and 0 points for errors. Five-point Likert graded questions were divided into five levels: “strongly disagree”, “disagree”, “unsure”, “agree” and “strongly agree”. If the question was positive, choose “very agree” to score 2 points, choose “agree” to score 1 point, choose “uncertain” or “disagree” or “very disagree” to score 0, and vice versa. Further, the score of one question for food classification was 2 points, which was divided into 10 questions, with 0.2 points for each question and answer and no score for wrong answers.

### 2.2. The Validation of NLAI-L

#### 2.2.1. Sample Size

According to the exploratory factor analysis, it was considered sufficient to perform reliability and validity tests when the number of the sample size was five to ten times the number of questions [10]. Boomsma [11] recommended that the sample size for confirmatory factor analysis should not be less than 200. Some research points out that a sample size of about 300 is ideal, and a sample size of about 150 is sufficient [12]. A total of 397 participants were used for the validity test, so the sample size in our study was acceptable.

#### 2.2.2. Validity Test

(1) Content validity

Experts in relevant fields were invited to evaluate the questionnaire questions. There were six assessment experts included. Content validity was evaluated using the content validity index (CVI) and content validity ratio (CVR).

When testing the content validity index (CVI), experts filled out the five-point Likert scale according to relevance and rated each question. The scale was set as follows: (1) very irrelevant, (2) irrelevant, (3) general relevant, (4) relevant, (5) very relevant. Calculate the item content validity index (I-CVI) of the project by dividing the total number of experts who selected options four and five by the total number of experts. When the entry I-CVI ≤ 0.78, consider revising or adjusting [13]. The scale validity index (S-CVI) was calculated as the average of I-CVI. The content validity ratio (CVR) was calculated using the Lawshe formula to measure the importance of the survey items [14]. The experts defined the purpose of the questionnaire and provided the relevant operational definitions of the content questions. Each question was given to experts based on a three-point Likert special scale, and each question was rated respectively. Entries are (1) unnecessary, (2) useful but unnecessary and (3) necessary. Entries with CVR < 0.60 are substandard. Revise projects with CVR between 0.60 and 0.80.

Pearson correlation coefficient was computed to analyze the correlation between NL questions and the total score of each dimension. When *p* < 0.05, it indicates that the correlation between questions and dimension scores was statistically significant. When r > 0.2, the correlation is good [15].

(2) Construct validity test

Exploratory factor analysis (EFA) is used to establish the theoretical framework for each dimension. Kaiser–Mayer–Olkin (KMO) and Bartlett’s spherical test is used to check the suitability of EFA.KMO > 0.5, and *p* < 0.05 in Bartlett’s test of sphericity suggests the feasibility of EFA [16].

Confirmatory factor analysis (CFA) is used to verify the theoretical framework established by EFA and to test the authenticity and appropriateness of the construct validity. In this study, the overall model adaptation index refers to the Root Mean Square Error of Approximation (RMSEA). If RMSEA is less than 0.08, the model is acceptable, and if RMSEA is less than 0.05, the model fits well. The larger the CFI and TLI and the smaller the RMSEA, the better the model [17]. The reasonable criteria of other indices were as follows: the Comparative Fit Index (CFI) >0.90, the Adjusted Goodness of Fit (AGFI) ≥ 0.80 [18,19] and the ratio of χ^2^ to the degree of freedom (χ^2^/*df*) <3 [20].

#### 2.2.3. Reliability

Cronbach’s α coefficient is the most commonly used measurement method at present. Cronbach’s α > 0.7 indicates that the questions in the total scale are consistent [21].

Split-half reliability is also often used to test the consistency of the scale across dimensions [22]. The questions of each dimension of the scale are divided into odd and even halves, and the correlation between the scores of the two parts is calculated. The calculation is to be corrected and restored by Spearman–Brown formula. 

### 2.3. The Application of NLAI-L

To evaluate the NL level of lactating women in China and analyze the potential influencing factors, 622 lactating women were included in our study. The on-site investigation and online survey were carried out. The inclusion and exclusion criteria were the same as that mentioned above.

### 2.4. Data Analysis

The CFA was carried out using software AMOS23.0 (SPSS Inc., Chicago, IL, USA). The analysis for exploring potential factors was carried out using R4.2.2 (R Foundation for Statistical Computing; http://www.rproject.org (accessed on 10 February 2023)). Multiple linear regression model was performed to determine the factors related to NL levels of Chinese lactating women. To exclude the influence of confounding factors to some extent, factors with *p* < 0.10 in the one-way analysis were included in the multivariate analysis model. The significance level of the statistical test was 0.05 on both sides. 

## 3. Results

### 3.1. The Dimension and Topics of NLAI-L

The framework of NLAI-L was ultimately made up of three dimensions: knowledge dimension, behavior dimension and skill dimension. Each dimension consisted of four, one and four topics, respectively (shown in Table 2).

### 3.2. Demographic Characteristics in Confirmatory Study

A total of 397 valid questionnaires were used for confirmatory study. The demographic characteristics are shown in Table 3. The average age was 30.6 ± 4.0 years old, of which 12.3% were over 35 years old. Additionally, 18.7% of them were overweight or obese according to pre-pregnancy BMI, while 37% were overweight or obese according to post-pregnancy BMI. In total, 93.7% were ethic Han. As well, 23.2% had other characteristics, as shown in Appendix A.

### 3.3. Content Validity

#### 3.3.1. I-CVI and CVR

The I-CVI ranged from 0.67 to 1.00 in our study. In behavior dimension, the I-CVI was 1.0, referring to satisfactory content validity, while the I-CVI of 0.67–1.0 in knowledge dimension and skill dimension noted the limited content validity (Table 3).

#### 3.3.2. Pearson Correlation Coefficient

The correlation between knowledge dimension, skill dimension and overall instruments of aggregate validity and discrimination validity is 0.89 and 0.71, respectively, which means that there is a high correlation. The skill dimension is 0.65, close to 0.70, showing a moderate correlation, and the overall aggregate validity is acceptable. The correlation between dimensions is between 0.36 and 0.41, indicating that the discriminant validity is within the acceptable range (Table 4).

### 3.4. Construct Validity Test

#### 3.4.1. Exploratory Factor Analysis

EFA was carried out on the three dimensions of knowledge, behavior and skills. KMO was 0.82, 0.73 and 0.64, respectively, and Bartlett’s spherical test values were all statistically significant, which implies the feasibility of EFA. In the knowledge dimension, four factors were extracted and accounted for 38.87% of the cumulative explanation. They represented the first part of feeding knowledge, the second part of feeding knowledge, nutrition and health knowledge and food and nutrition knowledge. Items loaded between 0.23 and 0.79 in the dimension. In the behavior dimension, only one factor was obtained, explaining 35.30% of the total variance. It was named lifestyle and eating behavior. Items loaded between 0.50 and 0.70 in the dimension. In the skill dimension, four topics were identified, which made up 59.22% of the total variance. They were named interpretation of nutrition labels, nutrition information acquisition and decision-making, weight management for infants and weight management for lactating women. All the factor loadings were acceptable.

#### 3.4.2. Confirmatory Factor Analysis

The results are shown in Table 5. The GFI of the overall NLAI-L was 0.81, close to the nominal value of 0.9. RMSEA had a value of 0.057, less than 0.08. Each index was in the acceptable range. 

### 3.5. Reliability and Validity

The Cronbach’s α coefficient of the whole instrument is 0.84 and the Spearman–Brown coefficient is 0.74, indicating that the reliability of NLAI-L is good. The Cronbach’s α coefficient of each dimension is between 0.59 and 0.79, and the Spearman–Brown coefficient is between 0.57 and 0.65, as shown in Table 6.

### 3.6. Assessing Nutrition Literacy for Lactating Women in China and Its Related Factors

#### 3.6.1. The NLAI-L Scores of Chinese Lactating Women

Based on 622 participants recruited for application, the full score of the scale was 76, and the mean score of the study subjects was 46.0 ± 9.3, with a maximum score of 74 and a minimum score of 16.6. A total of 353 women scored over 60%, accounting for 56.8%. The average score of knowledge dimension was 29.6 ± 5.7, the average score of behavior dimension was 5.0 ± 2.5 and the average score of skill dimension was 11.4 ± 3.5. Other basic characteristics and scores are shown in Table 7.

#### 3.6.2. The Potential Factors Influencing Nutrition Literacy of Chinese Lactating Women

**Figure 2:**
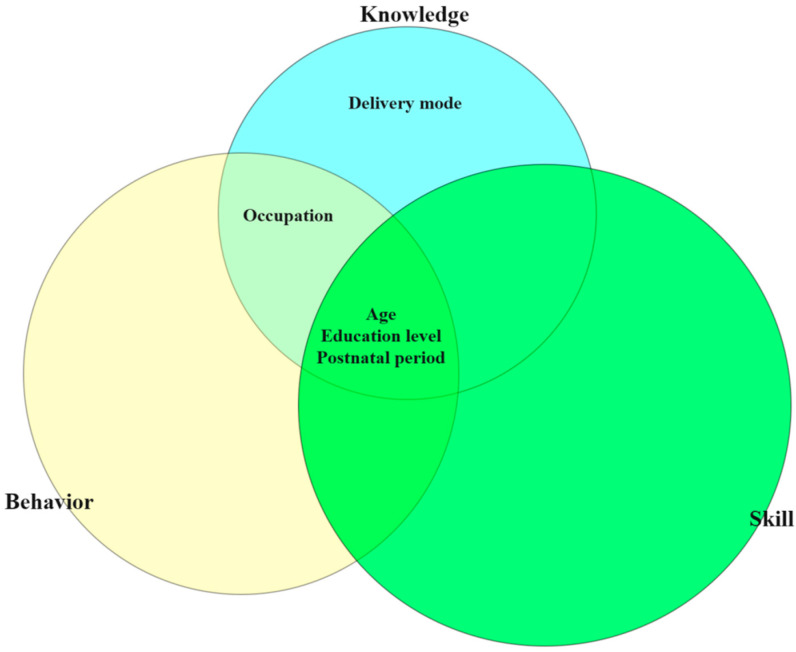
Venn diagram of influencing factors of NL scores in Chinese lactating women.

Univariate and multivariate analyses were used, respectively. As for overall scores, the influencing factors in the multivariate analysis include age and education level. In the knowledge and behavior dimension, scores were related to age, education level, postnatal period and occupation. As for skill dimension, lactating women younger than 25, with high school education or below, were housewives, and within 3 months postpartum had the lowest scores except for lactating women >24 months postpartum (Table 8). The Venn diagram shows associations between influencing factors and NL scores across all three dimensions (Figure 2).

## 4. Discussion

In this study, an effective and reliable instrument for evaluating the NL of Chinese lactating women (NLAI-L) was established qualitatively and quantitatively. In terms of content, NLAI-L includes three dimensions of knowledge, behavior and skills; nine topics (the first part: feeding knowledge, nutrition and health, food and nutrition; the second part: feeding knowledge, diet and lifestyle, reading and understanding of labels, information acquisition and decision-making of nutritional information, infant weight management, breastfeeding mother weight management); and 38 questions in total.

The knowledge dimension contains 22 questions, which is the most among the three dimensions. For the content of knowledge dimension, it consists of four topics representing feeding knowledge 1, nutrition and health knowledge, food and nutrition knowledge and feeding knowledge 2. From the results of item analysis and exploratory factor analysis, the topic of feeding knowledge is divided into two parts. The first part is mainly a five-point Likert scale, which primarily evaluates the breastfeeding knowledge recognition of breastfeeding mothers, while the second part is mainly information through single-choice and multiple-choice questions focusing on the details of specific feeding knowledge. Among the topics of food and nutrition knowledge, questions focus on food sources of important nutrients during lactation and their health effects. Gibbs also stressed the importance of popularizing the effects of nutrients in food on disease and health outcomes in nutrition education [5]. Nutrition and health knowledge involve questions such as a balanced diet and avoiding tobacco and alcohol during breastfeeding, emphasizing the importance of a healthy lifestyle to maternal and fetal health. In the feeding knowledge, the questions listed emphasize the importance of breastfeeding during lactation, feeding skills and the timing and skills of supplementary feeding, which have important guiding significance for breastfeeding. 

For the behavior dimension, the research group modified the topics of the behavior dimension, divided the relevant questions of feeding behavior into the knowledge dimension and retained only the lifestyle and eating behavior part. The reason for this is that most of the lactating women’s children are not old enough to add supplementary food, so the questions of feeding behavior are more appropriate as the examination of feeding knowledge. The dimension question belongs to functional NL, and the questions of lifestyle and eating behavior cover different types of food intake, water intake during lactation, milk and dairy product intake and so on. This is reflected in other NL assessment instruments [23,24,25,26].

Four topics were revealed in the skill dimension, representing the interpretation of nutrition labels, nutrition information acquisition and decision-making, weight management for infants and weight management for lactating women. In terms of content, the questions in the skill dimension tend to be functional and critical NL. The part regarding the interpretation of nutrition labels mainly examines the ability of nursing mothers to cook and apply nutrition labels, including judging the nutritional composition of food, so as to make a reasonable choice of food. In most assessment instruments, reading nutrition labels is an important part of the investigation [5,23,27,28,29]. The part regarding weight management for lactating women involves the aspect of breastfeeding mothers’ BMI classification and postpartum weight recovery. Reasonable weight ranges play an important role in the long-term health of nursing mothers. The infant weight management part includes the length and weight measurement time of infants of different ages. The part regarding nutrition information acquisition and decision-making mainly examines whether lactating women can identify information from different sources, so as to obtain nutrition information and knowledge, and whether to apply knowledge to make nutrition decisions.

Generally speaking, NLAI-L has good content validity and construct validity, and the reliability of knowledge and skill dimensions is good, but the reliability of the behavior dimension is general, which is related to the small number of questions and individual differences in behavior dimension. Due to the great differences in crowd behavior, it is necessary to have a sufficient number of questions in order to measure accurately, but this will lead to too many questions in the questionnaire, take too long to answer all the questions and affect the quality of the overall answers in order to strictly control the length of the instrument. This time, we screened the representative behavior questions and gave up a certain degree of reliability. The instrument is constantly evolving and improving, and we will add behavior-related questions later as needed. In terms of content, the questions retained by NLAI-L cover functional, interactive and critical NL. From the perspective of research methods, we should first establish a theoretical framework and establish the core questions of the NL of lactating women, and there is a solid theoretical basis for dimensional division and compilation of questions. It is a multi-level assessment instrument.

The results in the multivariate linear model indicated that age, occupation, education levels and postnatal period were the main factors influencing the NL for lactating women, with each of them having a significant influence on at least one dimension of the NLAI-L. Lactating women younger than 25 years old were more likely to have lower levels of NL, which may be due to the fact that this group is more likely to have lower levels of education due to their earlier age during childbearing and lower levels of all NL due to social experience. Among the different occupations listed in the questionnaire, housewives appear to have lower scores in all dimensions. This suggests that housewives may have less contact with others, which may result in less access to relevant knowledge and less chance of being influenced by others. We also found that education level was one of the main factors in the NL in all dimensions. Similar to our results, an investigation on NL among the elderly in America showed that education level and age could be influencing factors for NL [30]. A study among Italian adults indicates that being young and having a low school education level were factors associated with low nutrition literacy [31]. Another study in Greece showed that education was positively associated with nutrition literacy [32]. The postnatal period was another factor influencing lactating women’s NL scores. We found that women who were within 3 months postpartum had significantly lower levels of NL except for those who were over 24 months postpartum. This may be because they had less time to learn about the process of feeding their infants, which leads to increased NL. For women who were more than 24 months postpartum, there is little practical significance in improving NL for lactating women. In addition, it should be noted that lactating women who give birth naturally have higher NL scores in the knowledge dimension. This may be because they are more aware of the disadvantages of a cesarean section, and the possibility of cesarean section for non-medical reasons may decline [33,34]. However, the hypothesis mentioned above needs to be verified by researchers in China after using NLAI-L to investigate the NL of people in a larger population.

Our study had some advantages. First of all, it was the first instrument to evaluate the NL of Chinese lactating women. We conducted reliability and validity tests in a sufficiently large sample size and obtained acceptable results. Secondly, the development of NLAI-L has a solid theoretical foundation. The core items were scientifically established with the participation of experts and references from Chinese Citizen’s Health Literacy—basic knowledge and skills.

However, there are some limitations to our study. This study has limitations on the diversity and number of samples. When the research was carried out for the first time, limited by the promotion conditions, the samples were mostly concentrated in the eastern part of China, and there may be a certain bias in the economic income and HL of people in different regions. The results of CFA need to be confirmed in a larger population sample. The overall reliability of the questionnaire is high (Cronbach’s α coefficient is 0.84), but Cronbach’s α coefficients of food intake types and nutrition information acquisition topics are lower (0.31 and 0.45, respectively). This is similar to the NL series instrument NLAI-L developed by Gibbs and others [5,35], which may be related to the small number of topic questions. [36]. NLAI-L is still preliminary, and the research group will improve the relevant items in the follow-up investigation. In addition, there is still some room for research on the relationship between NL level and lactation outcome. More surveys are needed to improve demographic information and combine dietary surveys and biochemistry to explore the correlation between NLAI-L and dietary intake and more nutritional indicators.

## 5. Conclusions

This study established an effective and reliable assessment instrument (NLAI-L) through qualitative and quantitative methods. It had good content validity, acceptable construct validity and excellent reliability and validity. Potential influencing factors for Chinese lactating women’s nutrition literacy included education level, occupation, age and postnatal period according to our survey with NLAI-L. The establishment of NLAI-L will provide an effective instrument for exploring the role of NL in health or disease and lay a foundation for the formulation of targeted nutrition intervention measures.

## Figures and Tables

**Table 1:** The core items of NL for lactating women in China.

Dimension	Topic	Items
Basic knowledge and concept	Nutrition concept	1. A variety of foods during breastfeeding, but not too much, to ensure a balanced and adequate nutrition is vital to the health of the mother and child.
		2. Lactating women should adhere to a balanced diet, moderate exercise and gradually return to the appropriate weight.
	Food and nutrition knowledge	3. Milk is rich in calcium that is easily absorbed, making it an ideal source of calcium during lactation.
	Feeding knowledge	4. Breast milk is the most ideal natural food for infants, and the first food for newborns should be breast milk.
		5. Exclusive breastfeeding is recommended for 6 months. Infant formula is only available when exclusive breastfeeding is not possible.
		6. Babies’ physiological weight loss can occur within a week of birth, usually less than 7% of birth weight, and recover to birth weight within 7–10 days.
		7. Complementary feeding starts with iron-rich pureed foods, following the principle of gradual progress from less to more, from thin to thick, from fine to coarse.
		8. Breastfeeding helps mothers recover to pre-pregnancy weight and reduces the risk of breast cancer, ovarian cancer and type 2 diabetes in mothers, and reduces the risk of infectious diseases and allergies in infants.
Lifestyle and dietary behavior	Lifestyle	9. Lactating women should avoid alcohol, tobacco, strong tea and coffee.
	Dietary behavior	10. Lactating women should increase their intake of foods rich in high-quality protein.
		11. Lactating women should consume enough vegetables and fruits every day, ensuring a daily vegetable intake of 500 g, with dark vegetables (green and red-yellow vegetables) accounting for more than 2/3.
		12. Lactating women should increase the intake of animal foods rich in vitamin A to an appropriate amount and eat animal liver once or twice a week.
		13. Use iodized salt and eat iodine-rich seafood, such as seaweed and nori, once or twice a week.
		14. Lactating women should increase their intake of milk, and the total amount of milk consumed should reach 500 mL per day.
		15. Lactating women should ensure adequate daily water intake and drink no caffeinated beverages.
	Feeding behavior	16. Newborns should start breastfeeding as early as possible after birth, with early contact, early sucking and early initiation of breastfeeding.
		17. Start complementary foods at 6 months of age and continue breastfeeding until 2 years of age or older.
		18. Daily vitamin D supplementation should be started about 2 weeks after birth; exclusively breastfed infants do not need calcium supplementation.
		19. Infants should be fed on demand for 6 months.
		20. Insist that infants suckle directly from the breast and not use bottles for indirect feeding of artificially expressed breast milk whenever possible.
Basic skills	Weight management	21. Regularly monitor infants’ physical indicators to pursue healthy growth.
	Supplementary food production	22. Infant complementary food should be made separately using safe, high-quality and fresh ingredients. Keep the production process clean and hygienic, maintain the original taste of food and minimize the use of sugar, salt and various condiments.
	Information identification and decision-making	23. Pay attention to food labels and choose packaged foods wisely.
	Nutrition information acquisition	24. Pay attention to nutrition information and be able to obtain, understand, identify and apply lactation nutrition information.

**Table 2:** The dimension and topics of NLAI-L.

Dimension	Topic	Questions
Knowledge	Feeding knowledge 1	9
	Feeding knowledge 2	5
	Nutrition and health knowledge	2
	Food and nutrition knowledge	6
Behavior	Lifestyle and eating behavior	6
Skill	Interpretation of nutrition label	4
	Weight management for lactating women	1
	Weight management for infants	2
	Nutrition information acquisition and decision-making	3
Total		38

**Table 3:** The CVI and CVR of each dimension and the whole NLAI-L.

Dimension	S-CVI	CVR	I-CVI
Knowledge	0.98	0.97	0.66–1.0
Behavior	1.0	1.0	1.00–1.0
Skill	0.97	0.93	0.66–1.0
NLAI-L	0.98	0.96	0.66–1.0

**Table 4:** Correlation between each dimension and NLAI-L.

Dimension	Knowledge	Behavior	Skill
Knowledge			
Behavior	0.41		
Skill	0.40	0.36	
NLAI-L	0.89	0.65	0.71

**Table 5:** Confirmatory factor analysis of NLAI-L.

Dimension	*χ*^2^/*df*	GFI	AGFI	RMSEA
Knowledge	1.55	0.93	0.91	0.037
Behavior	0.98	0.99	0.98	0.000
Skill	1.84	0.97	0.95	0.046
NLAI-L	2.28	0.81	0.79	0.057

**Table 6:** Reliability of NLAI-L and each dimension.

Dimension	Cronbach’s α	Split-Half Reliability	
		Spearman–Brown	Guttman Split-Half
Knowledge	0.79	0.65	0.64
Behavior	0.66	0.63	0.63
Skill	0.59	0.57	0.55
NLAI-L	0.84	0.74	0.74

**Table 7:** Characteristics of participants in the application.

Characteristics	*n* (%)	$\mathrm{Scores}\;(\overset{-}{X}\;\pm \;\mathrm{Sd})$
Pre-pregnancy BMI		
Normal	433 (69.6)	46.4 ± 9.0
Underweight	56 (9.0)	48.0 ± 10.8
Overweight	108 (17.4)	43.7 ± 8.9
Obesity	25 (4.0)	45.6 ± 12.0
Education level		
High school or lower	51 (8.2)	38.5 ± 8.4
College	116 (18.6)	43.0 ± 8.9
University	286 (46.0)	46.0 ± 8.9
Postgraduate and above	169 (27.2)	50.5 ± 8.3
Age		
≤25 years old	28 (4.5)	38.2 ± 11.9
25–30 years old	216 (34.7)	46.3 ± 10.0
30–35 years old	242 (38.9)	46.4 ± 8.6
>35 years old	121 (21.9)	46.5 ± 8.4
Postnatal period		
≤3 month	267 (42.9)	45.0 ± 9.2
3–6 month	102 (16.4)	47.9 ± 10.1
6–12 month	142 (22.8)	47.7 ± 9.0
12–24 month	55 (8.9)	48.6 ± 8.5
≥24 month	56 (9.0)	41.1 ± 7.5
Occupation		
Housewife	106 (17.0)	41.9 ± 10.4
Government agency	49 (7.9)	48.1 ± 9.7
Professional technician	208 (33.4)	48.3 ± 8.5
Office clerk or related personnel	73 (11.7)	48.1 ± 7.7
Business/services	88 (14.2)	45.5 ± 7.5
Others	98 (15.8)	43.8 ± 10.1
Ethnic groups		
Han	565 (90.8)	46.3 ± 9.2
Others	67 (8.2)	43.9 ± 10.5
Region		
Eastern China	559 (89.9)	46.2 ± 8.9
Middle China	40 (6.4)	43.8 ± 12.5
Western China	23 (3.7)	45.5 ± 12.2

**Table 8:** Potential factors influencing NL of lactating Chinese women.

Characteristics	β ^a^ in NLAI-L Score (95% CI)	β ^b^ in Knowledge Score (95% CI)	β ^c^ in Behavior Score (95% CI)	β ^d^ in Skill Score (95% CI)
Pre-pregnancy BMI				
Normal				
Underweight	2.25 (−0.17, 4.67)	1.46 (−0.06, 2.97)		0.28 (−0.64, 1.21)
Overweight	−1.80 (−3.67, 0.07)	−0.92 (−2.09, 0.25)		−0.64 (−1.36, 0.07)
Obesity	1.91 (−1.58, 5.40)	0.80 (−1.38, 2.98)		0.22 (−1.11, 1.55)
Education level				
High school or lower				
College	3.52 (0.59, 6.46)	1.89 (0.05, 3.73)	0.84 (0.00, 1.68)	0.86 (−0.26, 1.99)
University	5.42 (2.46, 8.37)	2.85 (1.01, 4.70)	1.26 (0.42, 2.10)	1.27 (0.14, 2.41)
Postgraduate and above	8.99 (5.77, 12.20)	4.57 (2.56, 6.58)	1.67 (0.75, 2.58)	2.69 (1.46, 3.93)
Age				
≤25 years old				
25–30 years old	5.17 (1.64, 8.71)	2.70 (0.46, 4.93)	1.04 (0.03, 2.05)	1.29 (−0.07, 2.65)
30–35 years old	5.49 (1.95, 9.03)	2.82 (0.58, 5.07)	1.02 (0.02, 2.02)	1.51 (0.15, 2.87)
>35 years old	5.31 (1.60, 9.02)	2.40 (0.03, 4.77)	1.16 (0.11, 2.21)	1.52 (0.10, 2.95)
Postnatal period				
≤3 month				
3–6 month	1.83 (−0.14, 3.80)	1.39 (0.15, 2.62)	1.46 (0.34, 2.59)	0.47 (−0.28, 1.23)
6–12 month	1.67 (−0.12, 3.45)	1.41 (0.29, 2.53)	1.54 (0.52, 2.55)	0.57 (−0.11, 1.25)
12–24 month	1.81 (−0.72, 4.35)	1.53 (−0.06, 3.12)	1.72 (0.27, 3.16)	0.43 (−0.55, 1.40)
≥24 month	−4.15 (−6.75, −1.56)	−1.36 (−2.97, 0.25)	−1.36 (−2.83, 0.11)	−2.15 (−3.14, −1.17)
Occupation				
Housewife				
Government agency	2.48 (−0.73, 5.68)	2.46 (0.45, 4.48)	2.55 (0.72, 4.38)	0.48 (−0.75, 1.71)
Professional technician	1.79 (−0.62, 4.19)	1.67 (0.15, 3.19)	1.67 (0.29, 3.05)	0.19 (−0.73, 1.11)
Office clerk or related personnel	1.84 (−1.00, 4.67)	1.68 (−0.11, 3.47)	1.84 (0.21, 3.47)	0.58 (−0.51, 1.67)
Business/services	1.49 (−1.04, 4.02)	1.20 (−0.40, 2.81)	1.24 (−0.22, 2.71)	0.38 (−0.60, 1.35)
Others	−1.31 (−3.81, 1.19)	0.23 (−1.35, 1.80)	0.37 (−1.07, 1.80)	−0.74 (−1.70, 0.22)
Ethnic groups				
Han				
Others	−1.90 (−4.26, 0.47)			
Delivery mode				
Spontaneous labor				
Others	−0.93 (−2.36, 0.49)	−0.99 (−1.89, −0.10)		

BMI, body mass index. ^a^ Variations in the multivariate regression include pre-pregnancy BMI, education level, age, postnatal period, ethnic groups. ^b^ Variations in the multivariate regression include pre-pregnancy BMI, education level, age, postnatal period, occupation, delivery mode. ^c^ Variations in the multivariate regression include education level, age, postnatal period, occupation. ^d^ Variations in the multivariate regression include pre-pregnancy BMI, education level, age, postnatal period, occupation.

## Data Availability

The datasets used during the current study are available from the corresponding author upon reasonable request.

## Appendix A

**Table A1:** Demographic Characteristics of Respondents in Confirmatory Study.

Variables	N = 397
Age	
<25	19 (4.8)
25–30	198 (49.9)
30–35	131 (33.0)
>35	49 (12.3)
Ethnic group	
Han	372 (93.7)
Minority	25 (6.3)
Occupation	
Unemployed	18 (4.5)
Housewife	142 (35.8)
Office clerk or related personnel	47 (11.8)
Business/services	45 (11.3)
Professional technicians	84 (21.2)
Others	61 (15.4)
Pre-pregnancy BMI	
Underweight	70 (17.6)
Normal	253 (63.7)
Overweight	55 (13.9)
Obesity	19 (4.8)
Education level	
Senior high school and below	94 (23.7)
College	111 (28.0)
University	142 (35.8)
Postgraduate and above	50 (12.6)
Post pregnancy BMI	
Underweight	27 (6.8)
Normal	223 (56.2)
Overweight	116 (29.2)
Obesity	31 (7.8)
Height(cm)	161.2 ± 5.0
Pre-pregnancy weight (kg)	55.7 ± 9.9
Postpartum weight (kg)	60.0 ± 10.3

Mean (standard deviation) for the continuous variables, N (%) for the categorized variables.
